# An integrative review of research evaluating organisational culture in residential aged care facilities

**DOI:** 10.1186/s12913-023-09857-y

**Published:** 2023-08-14

**Authors:** Kate Churruca, Emma Falkland, Maree Saba, Louise A Ellis, Jeffrey Braithwaite

**Affiliations:** Centre for Healthcare Resilience and Implementation Science, Australian Institute of Health Innovation, Macquarie University, Level 6, 75 Talavera Road, North Ryde, NSW Australia

**Keywords:** Organisational climate, Aged-care staff, Safety culture

## Abstract

**Background:**

Evidence suggests that the culture of healthcare organisations, including residential aged care facilities (RACFs), is linked to the quality of care offered. The number of people living in RACFs has increased globally, and in turn, attention has been placed on care quality. This review aimed to identify how organisational culture is studied, sought to elucidate the results of previous studies, and aimed to establish what interventions are being used to improve organisational culture in RACFs.

**Methods:**

We employed an integrative review design to provide a comprehensive understanding of organisational culture. Five academic data bases were searched (Ovid Medline, Scopus, PsycInfo, CINAHL, Embase). Articles were included if they were empirical studies, published in peer reviewed journals in English, conducted in a RACF setting, and were focused on organisational culture/climate.

**Results:**

Ninety-two articles were included. Fifty-nine studies (64.1%) utilised a quantitative approach, while 24 (26.0%) were qualitative, and nine used mixed methods (9.8%). Twenty-two (23.9%) aimed to describe the culture within RACFs, while 65 (70.7%) attempted to understand the relationship between culture and other variables, demonstrating mixed and indeterminate associations. Only five (5.4%) evaluated an intervention.

**Conclusions:**

This review highlights the heterogenous nature of this research area, whereby differences in how culture is demarcated, conceptualised, and operationalised, has likely contributed to mixed findings. Future research which is underpinned by a sound theoretical basis is needed to increase the availability of empirical evidence on which culture change interventions can be based.

**Supplementary Information:**

The online version contains supplementary material available at 10.1186/s12913-023-09857-y.

## Introduction

Increasingly, the culture of a healthcare organisation is recognised as intrinsically linked with its performance and quality of care [1, 2]. Systematic reviews have found that an organisational culture characterised by effective leadership, relationships and teamwork is associated with better patient outcomes, including reduced mortality, better organisational performance and improved satisfaction [3, 4]. The literature on culture, however, contains considerable heterogeneity in terminology and definitions used, methods for studying it, and theoretical underpinnings [5]. This variability challenges our ability to make sense of, address, and improve culture, guidance that is sorely needed by policymakers and healthcare managers whom regularly highlight cultural issues as contributing to shortcomings in care quality [6].

The number of older people living in residential aged care facilities (RACFs, also referred to as nursing homes, skilled nursing facilities or assisted living facilities) has increased over the past decade in Australia, as in many other countries [7]. Over this time, the people living in these facilities have also become older, frailer and sicker [8]. There has been a growing attention to problems in the treatment of older people and the quality and safety of care provided within RACFs, which have often been attributed to “cultural failings” [9]. Distinct from acute healthcare settings, RACFs are *homes* for people requiring assistance with activities of daily living, whom have diverse psychological, social and spiritual needs [10], and many of whom have cognitive or physical impairment. In effect, this places additional and unique expectations on care quality, and on what sorts of culture(s) might contribute to it.

Research on organisational culture in RACFs has increased over the past few decades, though reviews have been more limited and focused specifically on culture as it relates to the safety of care delivered to residents [11, 12]. This concept of safety culture is studied frequently in hospitals, where measurement is used for regulatory and accreditation purposes, quality improvement, research, and as a proxy to monitor patient safety [13, 14]. Although the concept has relevance to aged care, it focuses on organisational norms, values and behaviours that explicitly relate to clinical and patient safety, rather than the more diverse and holistic aspects of care quality within RACFs. A synthesis of research on safety culture in RACFs, therefore, provides only a partial representation of the types and aspects of cultures existing in these settings, and how culture is related to care quality, including in outcomes for both residents and staff.

### Aim and research questions

The aim of this review is to integrate diverse studies of organisational culture in aged care facilities. The following research questions were formulated:

1. How is organisational culture being studied and conceptualised in RACFs?
2. What are the primary aims and results of studies on organisational culture in RACFs?
3. What interventions are being designed and used to improve organisational culture in RACFs?

## Methods

An integrative review design was chosen because it can provide a comprehensive understanding of a phenomenon, providing insights into both the current state of knowledge and future research directions related to theory, methodology and practice [15, 16]. Moreover, the approach allows for integration across a potentially fragmentary research field, which our experience and that of others has suggested could be the case for this area [15].

Research literature and websites were searched for reviews or review protocols on this subject. With none identified, a protocol was developed to fill this gap, and subsequently registered on the Open Science Framework (OSF) Registries on 8 April 2022 (osf-registrations-evbkt-v1). Reporting of the review results followed the Preferred Reporting Items for Systematic Reviews and Meta-Analyses extension for Scoping Reviews (PRISMA-ScR) [17], where appropriate, as there are no such standards for integrative reviews currently available.

### Preparing the guiding question

In preparing the guiding question, we drew from our experience in psychology and health services research. Literature in the latter area often conflates concepts of climate and culture [5, 18], despite numerous researchers suggesting conceptual or methodological nuances between them [19, 20]. As such, both were included in the review, and differences in terminology extracted to inform a synthesis. Only empirical research was included as relationships between culture and care quality in RACFs were of interest, with studies needing to include a description of how data (primary or secondary) was collected and analysed. The following inclusion criteria were devised:

1. Full articles published in English in a peer-reviewed journal.
2. All types of empirical studies (quantitative, qualitative, mixed methods; use of primary or secondary data).
3. Setting is residential aged care– the empirical study is being conducted in a RACF (e.g., nursing home, long term care facility, assisted living, skilled nursing facility) or collecting data from those working/living in facility.
4. Focused on understanding organisational culture/climate – the study is examining or assessing organisational culture, or some facet thereof (e.g., safety culture), as the focus of the study (e.g., not a secondary outcome measure).

The exclusion criteria were:

1. Studies published in a language other than English, not published in a journal (e.g., book chapters) or not a full article (e.g., conference abstracts).
2. Non-empirical articles (e.g., commentary, literature review).
3. Studies not in residential aged care or where responses from those within RACFs cannot be separated out.
4. Studies not focused on understanding organisational culture as the main aim. This involved the exclusion of studies that measured culture as a secondary outcome, articles with a methodological focus (e.g., validating an organisational culture survey) or investigating “culture change” but not evaluating culture.

### Searching the literature

The search strategy was informed by past systematic reviews on organisational culture and residential aged care facilities [3, 10, 12], and consultation with a research librarian. Five databases were searched (Ovid Medline, Scopus, PsycInfo, CINAHL, Embase) using the keywords listed in Fig. 1 in April 2022. The full search for Ovid Medline including MESH terms is in Additional File 1.

**Fig. 1:**
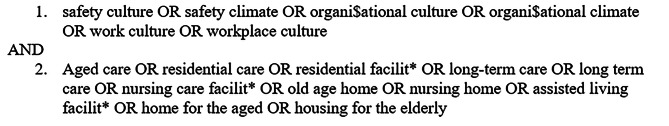
Search terms for review

References were downloaded into Endnote where duplicates were removed. Citations and abstracts were then exported to the online collaborative systematic review platform RAYYAN for screening [21]. Title/abstract screening was completed independently by KC and a research intern (HN) who resolved any disagreement through discussion. Full text review was then also conducted by KC, EF, and MS independently, with reasons for exclusion documented.

### Data extraction process

Data from included studies were extracted into a custom template developed in Microsoft Excel. In addition to the findings of studies, we sought to capture methodological and theoretical issues in data extraction given results are to some extent a function of decisions made regarding methods and theory [5]. The template captured the data items in Table 1.

**Table 1:** Data items to be extracted

Research question tapped	Data item
1. How is organisational culture being studied and conceptualised in RACFs?	a. Where and when are studies being published? b. What terms, concepts and theories are being used to conceptualise culture? c. What are the methods? d. What are the tools being used? e. Who are the participants?
2. What are the primary aims and results of studies on organisational culture in RACF?	a.What is the primary purpose of the study? b.What are the results in terms of how culture in RACFs is described? c.What other variables are examined in relation to culture? d. What associations with organisational culture are found?
3. What interventions are being used to improve organisational culture in RACFs?	a.What is the rationale for the intervention? b.What does it target? c.What are its main outcomes?

### Critical appraisal of individual sources of evidence

The Quality Assessment for Diverse Studies (QuADS) tool [22] was used to appraise the included studies because it provides a single set of items applicable across qualitative, quantitative, mixed and multiple method studies, and, befitting an integrative review on the subject of organisational culture, includes an attention to theory as a focus. It has 13 domains that cover issues of methodological and evidence quality (e.g., study design is appropriate to address the research question), as well as the quality with which studies are reported (e.g., strengths and limitations critically discussed). Each domain is scored from 0 to 3, with higher scores indicating higher quality.

### Synthesis of results

Data items were coded to facilitate synthesis. The primary purpose of each study was classified into one of three categories: describing culture, exploring relationships with other variables, or an intervention to improve culture [13]. Findings were inductively coded within these categories to identify common themes in descriptions of culture in RACFs, factors associated with culture, and the basis for, and results of, interventions. To illustrate this process, for factors associated with culture, this involved descriptively summarising the results of each study in a few keywords based on the constructs studied, then reading through to identify commonalities across codes and classify these into broader themes. For example, studies looking at adverse events, urinary tract infections, and falls were all classified under “Clinical outcomes and adverse events”. Data items were synthesised in tabular and narrative form to highlight frequencies of study types and broad themes and trends in the research findings.

## Results

**Fig. 2:**
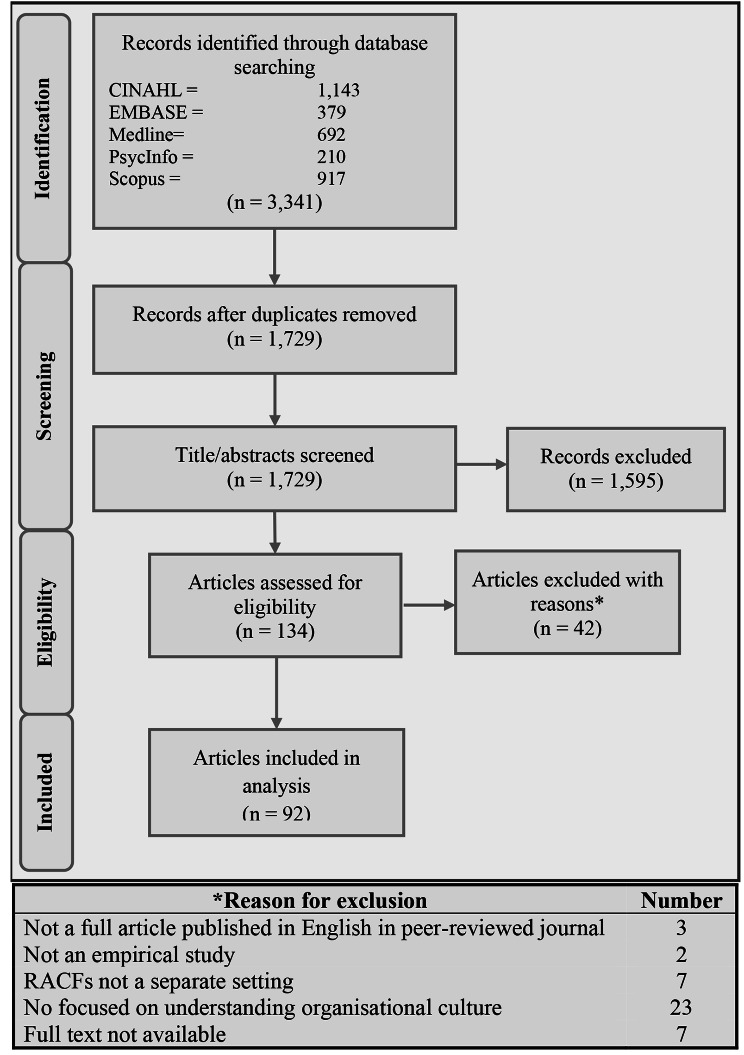
PRISMA flow diagram for the process of study selection

Searching the five academic databases resulted in 3,341 citations, which was reduced to 1,729 by removing duplicates. Title and abstract screening resulted in the exclusion of 1,595 citations, leaving 134 articles for full-text review. During this process, a further 42 articles were excluded with reasons documented, leading to a final set of 92 included articles (Fig. 2). Data extraction information for included studies is provided in Additional File 2.

### RQ1: how is organisational culture being studied and conceptualised in RACFs?

The included studies were published from 2001 onwards, with 2021 the year with the most articles published (n = 12, 13.0%). Research conducted in the United States made up the largest proportion of the dataset (n = 41, 44.6%), followed by Australia (n = 15, 16.3%) and Norway (n = 8, 8.7%); four studies were conducted (4.3%) in each of Canada, China and the Netherlands, three (3.2%) in Sweden and the United Kingdom, and two (2.2%) in New Zealand and multiple countries (New Zealand and the United Kingdom; Australia, Norway and Sweden). Single studies were conducted in Belgium, Finland, France, Ireland, South Korea, and Taiwan.

Most studies (n = 69, 75.0%) exclusively used the term “culture” to distinguish the conceptual focus of the study, while 16 (17.4%) concerned themselves with “climate” and seven (7.6%) used both, either drawing out their nuances or interchanging them. Many articles (n = 66, 71.7%) provided a description or definition of what they meant by the term organisational culture (or climate), though more explicit reference to a conceptual framework or theory about culture was less common (n = 23, 25.0%). Schein’s three-layer conceptualisation of culture [23] encompassing artifacts, values and underlying assumptions was the most widely-used framework, with the Competing Values Framework (CVF) [24] the only other recurring approach. Fifty-two studies (56.5%) focused on a specific type or aspect of the organisational culture within RACFs, with “safety culture” by far the most frequent of these (n = 33, 35.9%), followed by “safety climate” (n = 6) and “treatment culture” (n = 3).

The majority of studies (n = 59, 64.1%) utilised a quantitative approach, while 24 (26.0%) were qualitative, and nine studies used mixed methods (9.8%). Surveys were the most common method of data collection (either quantitative or mixed methods), reported in 67 studies (72.8%). In these studies, the nursing home version of the Agency for Healthcare Research and Quality (AHRQ) Surveys on Patient Safety Culture [25] was used most often (n = 20, 21.7%), though seven studies used the hospital version. Nine studies used tools based on the CVF, and four articles from the same team of authors team used the Veterans’ Affairs Community Living Center (CLC) Employee Survey of Attitudes about Resident Safety [26]. Twenty-seven studies (29.3%) assessed culture in residential aged care using interviews/focus groups, while 21 studies (22.8%) involved observations; seven studies that used both interviews and observations together had an overarching ethnographic approach.

The number of RACF sites included in a study was not always clear; where reported, this ranged widely, from a single site—typically studied in richer detail through ethnography e.g., [27] or a longitudinal survey e.g., [28]—through to a survey study of more than 4,000 facilities that looked at the association of nursing home culture with other administrative datasets [29]. Sample sizes and types of participants also varied. Some studies involved a mix of clinicians, or exclusively management or nurses, while others sampled from all aged care staff. In 13 studies (14.1%), residents or their families were participants, providing their perspectives on the organisational culture and the quality of care delivered within RACFs. For example, an Australian study used a survey of next-of-kin to understand the degree of family involvement in care [30], and in an ethnographic study from Norway, Nakrem [31] interviewed 15 residents about their lives in the nursing home.

### RQ2: what are the primary aims and results of studies on organisational culture in RACF?

The primary purpose of each study was classified into one of our three categories. Twenty-two (23.9%) aimed to describe the organisational culture within RACFs, while 65 (70.7%) attempted to understand the relationship between culture and other variables, and only five (5.4%) evaluated an intervention to improve culture. Findings across these different types of studies are summarised below.

#### Results of studies describing organisational culture in RACFs

Among studies aimed at describing organisational culture in RACFs, 11 (12.0%) were quantitative, six were qualitative and five used mixed methods. Despite variability in methodological approach, there were a few commonalities in the scope and findings of these articles. Challenges with leadership [32], resourcing and staffing [30], and the routinised, task-focused nature of care were highlighted as issues in the organisational culture of multiple studies [33]. Studies specifically investigating safety culture generally found it to be poor [34], particularly compared with studies of hospitals [35, 36], or home-based aged care [37], though one study suggested a more mixed picture in this regard [38]. Perceptions of safety culture also differed between professional groups within RACFs, with two studies finding that leaders held more positive views than did other staff [39]. Generally descriptive studies indicated that findings would or could be used as a basis for improving culture [34], with two ethnographic research studies explicitly treating the approach to data collection as a means to work with facilities on culture change [40, 41]. For example, Deutschman [40] took a methodological approach that involved documenting surprising or noteworthy social processes during observations, discussing them with informants, forming more general hypotheses and then confirming these to reach the level of underlying assumptions. Her insights related to care home routine, leadership, recruitment, role-modelling, and staff and resident satisfaction, suggesting that facilities should focus on discussing these issues or assessing these constructs as a starting point for attempting culture change.

#### Results on the relationship between organisational culture and other aspects of care

The studies with a primary purpose of examining relationships between culture and other variables generally fell into one of two subcategories: investigating predictors of culture (n = 15, 16.3%) or the effects of culture on other processes and outcomes (n = 49, 52.3%); that is, culture was either treated as the outcome or the input to other factors within residential aged care. For the former, all studies were quantitative, and the inputs to culture were typically staff or organisational factors. Characteristics of residential aged care staff that were found to contribute to culture—or at least their perceptions of culture—included demographics [42], professional background [43], and length of employment [44]. Organisational characteristics that were found to influence culture within RACFs included leadership style, accreditation [29], staff turnover rates [45], readiness to change [46], and the organisation of work tasks [47], as well as broader structural factors such as geographic location or number of beds [48].

Among the 49 studies looking at the effects of organisational culture on other processes or outcomes within residential aged care, most were quantitative (n = 31, 62.3%) with 17 qualitative studies (35.0%) and a single mixed method study. The factors considered in these studies were classified inductively into the broad categories in Table 2. Across these categories, research findings were most consistent for a positive relationship between organisational culture and staff outcomes such as job satisfaction and organisational commitment. Evidence was also supportive of a relationship between organisational culture and more psychosocial aspects of residents’ care, such as person-centred care and resident wellbeing. Some qualitative studies in this category examined how aspects of organisational culture contributed to humanistic treatment and residents’ experiences. There were also consistent findings that aspects of organisational culture (resident-centredness, teamwork) were associated with how psychotropic medicines were used in RACFs. For the most part though, factors were heterogenous within categories and the results were often inconclusive.

**Table 2:** Findings on the relationship between organisational culture and other processes and outcomes

Category	Number* and types of studies	Summary of findings
Ratings of care quality, person-centred care, and resident wellbeing	n = 16 including six qualitative and 10 quantitative survey studies	Six survey studies found some positive associations between quality of care (Government rated, researcher-observed, self-report) and aspects of culture. Three qualitative studies highlighted contextual nuances, but also found support for a relationship between organisational culture and quality of care. Some dimensions of patient safety culture were linked with the delivery of person-centred care, with cultural factors influencing person-centred care through a processing of “othering” residents. Resident satisfaction was also associated with facilities having a culture of companionate love. Finally, two qualitative studies examined how cultures of aged care delivery impeded residents’ capacity for autonomy, and how dependency culture could potentially lead to their mistreatment.
Clinical governance and care processes	n = 15 including eight qualitative, one mixed-methods, and six quantitative	Six studies identified a relationship between organisational culture and the use of psychotropic medicines in RACFs. Of these, the two quantitative studies found that a resident-centred culture was associated with lower use, while the four qualitative studies, all authored by the same research team, showed staffing, managerial expectations, and teamwork as influencing administration. Two quantitative studies found a negative relationship between safety culture and the use physical restraints, and another found it was associated with successful discharge planning for post-acute care residents. Five other qualitative studies linked aspects of culture within RACFs with the transition to palliative care for dying residents, antibiotic prescribing, continence care, and use of feeding tubes. No relationships were found between organisational culture or team climate and quality management at a facility ward level.
Staff outcomes and feelings about work	n = 11 studies, with one qualitative interview study, 10 quantitative survey studies	Seven studies found significant positive associations between organisational culture—or specifically ethical climate, culture of companionate love, safety climate—and aged care staff’s feeling, attitudes and behaviour related to work (e.g., job satisfaction, organisational commitment, willingness to stay). In another study, employee morale was also positively associated with an organisational culture orientated toward learning. Two studies found significant associations between organisational climate and staff’s self-efficacy in caring for residents with dementia. The sole qualitative study identified a culture of care within RACF that led staff to prioritise resident safety over their own.
Clinical outcomes and adverse events	n = 9 quantitative survey studies	Three studies found no associations between culture and adverse events, catheter-associated urinary tract infection and pressure ulcers. One study found a more positive patient safety culture among upper management was associated with less resident falls, while another with certified nursing assistants found the opposite, and also found no relationship with pressure ulcer rates. In a further two studies, higher safety culture/climate scores generally or along specific dimensions, corresponded with reduced risk of catheter use, major injuries from falls, pressure ulcers, and urinary tract infections. RACFs having a higher level of communication openness, a dimension of safety culture, also had higher rates of residents with dementia dying in the facility, as opposed to hospital. Finally, a study on a culture of companionate love found it was associated with less trips to the emergency room, but not with patient weight gain or ulcers.
Organisation-level processes and issues	n = 8 studies with two qualitative studies and six quantitative	Two studies investigated relationships between culture and staff turnover, finding mixed results, with differences between type of staff, and the levels of communication openness within the facility. A culture orientated toward learning had positive relationships with some organisational performance variables, and a culture of companionate love was positively associated with teamwork. Safety culture was negatively associated with perceptions of obstacles to event reporting, and climate dimensions of work pressure and innovation significantly predicted organisational readiness for change. One qualitative study identified organisational culture as differing in high versus low-teamwork facilities, with a more positive culture associated with higher teamwork. The other demonstrated how cultural orientations (“above and beyond,” “pushing back”, “engineering out”) provided a means of justifying different responses to regulation.

* Some studies examined factors from more than one category; study frequencies will not sum to total

One quantitative survey study that examined both climate and culture variables was unable to be classified discretely into focusing on predictors or outcome. It looked at whether organisational climate variables predicted safety culture in US nursing homes, finding four dimensions (efficiency, work climate, goal clarity, and work-stressed) were significant [49].

### RQ 3: what interventions are being used to improve organisational culture in RACFs?

Of the five studies evaluating culture change interventions in residential aged care, three were mixed methods, two involved purely qualitative data collection, and one was quantitative. These interventions varied in what they targeted, and in some the rationale for assuming a change in culture, or how the intervention specifically targeted culture, was not made clear. Two interventions provided education and training to staff to improve safety culture/climate; one of these studies specifically focused on the management of care transitions between hospitals and RACFs, but did not find improvements in survey scores among aged care staff [50]. The other had broader goals in upskilling staff and promoting a culture of continuous improvement, and while scores on the culture measure and other clinical outcomes improved, there was no control group comparison [51]. Two other studies focused on collaborative skills like teamwork and communication, both among staff and with residents and their families [52, 53]. Results for both studies indicated some improvements in culture, however, no controls were included. The final intervention had an explicit focus on positively reframing the RACF experience, finding improvements in cultural dimensions (e.g., empathy, task-orientation) over time compared with a control group [54].

### Quality appraisal

The overall average score on the quality appraisal tool for the studies included in this review was 25.3 out of a total possible score of 39. Studies tended to score highest on the seventh (mean of 2.8) and eleventh criterion (mean of 2.9), suggesting that vast majority of studies used appropriate methods of data collection and analysis, respectively. The lowest scoring criterion at an average of 0.04 was number 12, ‘Evidence that research stakeholders have been considered in research design or conduct’, indicating that there was typically no mention of research stakeholder inclusion informing the design or conduct of the study. “Theoretical or conceptual underpinning for the research” was also generally weak among included papers, with an average score of 1.3. In terms of study design, quantitative studies scored an average overall score of 25.9, mixed method approaches scored an average of 25.3, and qualitative studies scored an average score of 23.6.

## Discussion

Our integrative review of 92 studies of organisational culture in residential aged care found that research is generally concentrated among only a few countries, and approximately two-thirds of studies use quantitative designs. We identified heterogeneity in use of terminology and the facets of organisational culture focused on, as well as conceptual frameworks and tools. Nevertheless, findings on what culture looked like in RACFs demonstrated some recurrent themes including being task-focused or with a safety culture less advanced than in other healthcare settings.

The largest proportion of studies in our review—more than two-thirds—focused on understanding the relationship between the organisational culture of RACFs and other factors (e.g., clinical care processes, person-centred care, facility-level characteristics). Among these studies, evidence was most consistently supportive of an association between organisational culture and staff feelings about work such as job satisfaction or willingness to stay in the job, and for more psychosocial aspects of care, including residents’ wellbeing, satisfaction, and perceptions of quality. Although relationships were also found between culture and clinical care processes (e.g., psychotropic medicine use), these were less compelling; findings of an association between culture and clinical outcomes were even more equivocal.

Heterogeneity in findings is to some extent to be expected given the diversity of studies in this review; more than half investigated a more specific facet of the organisation’s culture such as safety or treatment culture, or a culture of companionate love. Moreover, a range of different survey tools and conceptual frameworks were utilised to understand and assess the concept. These difference in how culture was demarcated, conceptualised, and operationalised, in addition to the variation in what factors were studied in relationship to it, have likely contributed to the mixed findings.

Only five studies evaluated a culture change intervention, and the evidence for their effectiveness in aged care was generally limited. Such a small subset of studies dedicated to culture change interventions is somewhat surprising given the extensive rhetoric around culture change in the residential aged care space [57]. Moreover, a recent review of safety culture assessment in hospitals found a much more sizable proportion of approximately one-fifth of studies evaluated an intervention [13]. The result for the present review may in part be due to our inclusion criterion that studies focus on understanding culture as the main focus. In screening, this led to the exclusion of some articles that included the assessment of culture as a secondary outcome measure for their intervention e.g., [58], as well as studies of culture change as a concept or practice where the organisation’s culture was not assessed e.g., [59].

Another decision we made in formulating our review to include both studies of organisational culture and climate has yielded some further methodological considerations. Our results highlight a much greater preponderance of studies using the term “culture”, but this is not to suggest that culture *should* be the only term used, or that studies using “climate” are wrong. Many of the studies of organisational climate, or facets thereof, provided a clear definition of the construct that focused on individuals’ perceptions or experiences of the environment, which aligns with a common distinction made in the literature of culture as a deeper underlying construct, and climate its surface-level manifestation [20]. Climate studies in this review also overwhelmingly used surveys, with none exclusively employing qualitative methods. In this regard, Van den Berg and Wilderom [19] suggest that preferences for “culture” or “climate” reflect different research paradigms: sociological, qualitative and social constructionist compared with psychological and quantitative, respectively. This underscores the value of taking an integrative approach; it allowed us to synthesise findings from studies that use different research traditions, theories, and methodologies to focus on the largely intangible, social aspects of residential aged care workplaces. Finally, some studies used both climate and culture in their research, suggesting the potential additive value of incorporating the two concepts when they are appropriately theorised. For example, Sawan and colleagues drew upon Schein’s work to study the influence of organisational culture on psychotropic medicine use in RACF, considering the visible artifacts of culture [60], the invisible artifacts—the climate [61], and the basic underlying assumptions [62].

### Implications

The findings of this review have implications for research on organisational culture in residential aged care. First, there is a lack of research on interventions to improve organisational culture in this setting, with the few interventional studies identified typically not designed to conclusively demonstrate effectiveness. Studies investigating factors that influence culture were also in the minority, and for the most part examined variables that are not easily amenable to intervention, such as type of facility and staff demographics. This leaves us with limited empirical guidance upon which to base culture change interventions. Compelling findings regarding the influence of organisational culture on staff feelings about their work [28, 63−67], as well as trends suggesting relationships with perceptions of quality and care processes [28, 55, 56], nevertheless highlight the importance of understanding organisational culture, and how culture change can be achieved, within residential aged care.

Further research on factors associated with organisational culture in residential aged care is needed and such studies should ideally be underpinned by a solid theoretical basis for the conception of culture and any facets therein. Both through data extraction and the quality appraisal, it was apparent that many studies in this area do not have a theoretical or conceptual underpinning for the research. Moreover, despite the inherently interpersonal and contextual nature of culture, very few articles described involvement of research stakeholders in the design or conduct of the study. Indeed, the types of participants included to gain insight into culture were often limited, with quite a few studies relying upon only a single staff group (e.g., nurses, leadership), who make up only a portion of aged care workers and may differ in their perspectives of culture (climate) [35] or identify with a specific subculture [68]. On the other hand, some studies included residents and families alongside staff as participants, recognising that these groups may have insight into, or some degree of active involvement in, the culture within RACFs. Aged care facilities are not only a workplace dedicated to caring for these residents, but also their homes; our findings highlight residents, even those with dementia [27], or their families [56], can be involved in this sort of research to gain a fuller picture of the organisational culture and how it shapes their care experiences.

Finally, although safety culture/climate was the most commonly studied specific aspect of culture in RACFs, our dataset also included numerous articles focused on a “culture of companionate love” [28], a “person-centred climate” [56], or that drew out relationships between organisational culture and resident perceived aspects of care quality [31]. Research of this type recognises the diverse and holistic nature of residents’ care needs within RACFs and attempts to understand the role of organisational culture in meeting them; such research might be used to support the development of more person-centred guidelines for aged care in the future.

### Strengths and limitations

Our review had a comprehensive search strategy involving five academic databases and with input from a research librarian. Nevertheless, it is possible that some relevant papers were missed. Our inclusion criterion requiring culture as the focus excluded some articles that might have had informative results about culture change or the relationship between culture and other factors in RACFs. Alternatively, by focusing only on studies that sought to understand and evaluate culture, we were able to consider methodological and theoretical issues in how organisational culture is studied in this setting. We selected the QuADS quality appraisal tool to take account of the heterogeneity of studies and to focus on priorities in health services research such as stakeholder involvement, however, this has left us with limited scope to evaluate in detail the quality of certain types of studies (e.g., interventional, cross-sectional). The volume and diversity of articles identified suggests that the there is plenty of opportunity for more fine-grained review of a subset of this literature in the future, but that meta-analyses may be challenging, if impossible.

## Conclusion

The present review was designed to integrate the diverse range of research available pertaining to organisational culture in RACFs. The evidence presented highlights the largely heterogenous nature of this area of research, whereby differences in how culture is demarcated, conceptualised, operationalised, and measured, has likely contributed towards mixed findings. Of most importance, further research which is underpinned by a solid and sound theoretical basis is needed to increase the availability of empirical evidence on which culture change interventions in aged care facilities can be based upon.

### Electronic supplementary material

Below is the link to the electronic supplementary material.

Supplementary Material 1: Medline search

Supplementary Material 2: Data extraction

## Data Availability

The datasets used and/or analysed during the current study are available from the corresponding author on reasonable request.
